# Crystal Lattice Defects in Deuterated Zr in Presence of O and C Impurities Studied by PAS and XRD for Electron Screening Effect

**DOI:** 10.3390/ma16186255

**Published:** 2023-09-18

**Authors:** Agata Kowalska, Konrad Czerski, Paweł Horodek, Krzysztof Siemek, Mateusz Kaczmarski, Natalia Targosz-Ślęczka, Mathieu Valat, Rakesh Dubey, Krzysztof Pyszniak, Marcin Turek, Andrzej Droździel, Justyna Słowik, Jolanta Baranowska

**Affiliations:** 1 Physics Department, Maritime University of Szczecin, Wały Chrobrego 1-2, 70-500 Szczecin, Poland; 2 Institute of Physics, University of Szczecin, Wielkopolska 15, 70-451 Szczecin, Poland; 3 Institute of Nuclear Physics Polish Academy of Sciences, 31-342 Krakow, Poland; 4 Institute of Physics, Maria Curie-Skłodowska University in Lublin, pl. M. Curie-Skłodowskiej 1, 20-031 Lublin, Poland; 5 Faculty of Mechanical Engineering and Mechatronics, West Pomeranian University of Technology, al. Piastów 19, 70-310 Szczecin, Poland

**Keywords:** vacancies, dislocations, electron screening, DD fusion, positron annihilation spectroscopy, XRD

## Abstract

Low-energy nuclear reactions are known to be extremely dependent on the local crystal structure and crystal defects of the deuterated samples. This has a strong influence on both hydrogen diffusion and the effective electron mass. The latter determines the strength of the local electron-screening effect and can change the deuteron–deuteron reaction rates at very low energies by many orders of magnitude. In the present study, zirconium samples were exposed to various conditions and energies of deuteron beams using the unique accelerator system with ultra-high vacuum, installed in the eLBRUS laboratory at the University of Szczecin. Irradiated and virgin samples were investigated by means of the X-ray diffraction (XRD) and positron annihilation spectroscopy (PAS). While the first method delivers information about changes of crystal lattice parameters and possible production of hydrides accompanying the formation of dislocations that are produced during irradiation of the samples, the second one can determine the depth distribution of crystal defects, being especially sensitive to vacancies. The studied Zr samples were also implanted by carbon and oxygen ions in order to simulate the real situation taking place in nuclear reaction experiments and to investigate their influence on the kinetic of produced vacancies. The observed enhancement of the electron-screening effect in the deuteron fusion reaction at very low energies could be explained by formation of a high number of vacancies during the deuteron irradiation of samples. Possible carbon and oxygen impurities can affect this process in various ways by changing the depth distribution of vacancies and their diffusion, but they play only a minor role in the strength of the electron-screening effect.

## 1. Introduction

In sub-burrier nuclear reactions taking place in different metallic targets, the cross section is enhanced exponentially for lowering incident energies in comparison to the reaction cross section for bare nuclei [1]. The so-called electron-screening effect reduces the Coulomb barrier between two reacting nuclei mainly due to the presence of quasi-free electrons of the metallic environment, increasing the reaction rates in stellar nucleosynthesis and other fusion reactions by many orders of magnitude [2]. The experimental results of last years show that the reduction in the Coulomb barrier corresponding to the parameter known as screening energy can be of the order of 400 eV in the case of the deuteron–deuteron (DD) fusion reactions, while theoretical expectations give the value of about 100 eV [2,3,4]. This discrepancy was removed in the last experiments devoted to study the ^2^H(d,p)^3^H reaction under ultra-high vacuum conditions in an atomically clean Zr target [5]. We found that the observed enhancement of the DD cross sections at low energies can be explained by two different effects: a threshold resonance in the compound nucleus ^4^He [6] and the screening energy close to the theoretical prediction. The existence of the threshold resonance has been recently confirmed by observation of the electron–positron pair emission [7]. 

On the other hand, experiments carried out on a deuterated zirconium target using the UHV accelerator system showed that the enhancement due to the electron-screening effect changes with irradiation time probably due to the crystal lattice defects [8] induced by implanted deuterons and some target impurities, such as oxygen and carbon. It was demonstrated that the screening energy increased in the first two–three hours of irradiation and then decreased when the oxygen and carbon impurities accumulated at the target surface, which was monitored using Auger spectroscopy (see Figure 2 in [5]). The observed enhancement of the nuclear reaction at the initial stage of implantation was assigned to the deuterium trapping in the vacancies and localization of electrons in the crystal lattice defects due to local changes in the electron band structure of metals. 

This can be understood as a larger curvature of the electron valence band and—consequently—gain in the effective electron mass [9], leading to higher electron screening energy and significant enhancement in the reaction rate. According to the Thomas–Fermi model, the dependence of screening energy on the electron effective mass $m_{eff}$ can be described as follows:

(1) $$U_{TF}=\frac{e^{2}}{4\pi \varepsilon_{0}}k_{TF}=\frac{e^{3}}{4\varepsilon_{0}^{3/2}\hbar \pi^{2}}{\left(3\pi n\right)}^{1/6}\;m_{eff}^{1/2}$$

Here, *n* stands for the electron density and $k_{TF}$ is the Thomas–Fermi Wave number. 

A correlation between screening and electron effective mass was observed for example in the nonmetal–metal transitions in 0.5 atomic monolayer of magnesium on molybdenum [10]. The increase in the effective electron mass was in agreement with Mott–Hubbard transition and resulted in a value sixteen times higher than for free electrons.

Lately, a large influence of the occurrence of material defects on the screening energy was reported in [11]. Within the experiment, three different targets were used—carbon foil, cold-rolled palladium, and annealed palladium foils. Only in the case of cold-rolled palladium was the high electron screening potential detected. The authors conclude that the process of cold rolling increases the number of grain boundary defects into which hydrogen and deuterium atoms can be trapped. For comparison, the annealed sample did not give significant screening, since annealing reduces the number of structure defects. 

Apart from the dependence of material defects on the enhancement of nuclear reactions in metals, the structure of the crystal lattice also plays a significant role. An investigation performed on titanium targets [12] in inverse kinematics revealed that high screening potential was related to those Ti targets for which the XRD patterns matched cubic structure with deuterium on octahedral sites, while a small screening energy was linked to the dominant presence of the hexagonal pattern of Ti. Similar experimental evidence on the enhancement of the nuclear cross section was found in other deuterated fcc metals, i.e., Ni and Pd [1,2,5,13].

Hydrogen dissolution and diffusion and how the occurrence of impurities and crystal lattice defects may modify these processes have been a subject of wide theoretical research as well. Ab-initio calculations clearly show that creation of vacancies, in which hydrogen atoms can be trapped, lowers the overall free energy of the system, making this process energetically favorable [14]. Density Functional Theory (DFT) simulations for Ni demonstrate that mono and divacancies complex clusters in which up to twelve hydrogen atom can be segregated are characterized by stable configuration with high total biding energy [15]. This simulation technique applied to α-phase zirconium revealed that up to nine hydrogen atoms can be trapped in a single vacancy [16]. Within the same study, the role of carbon or oxygen impurities on hydrogen migration has been investigated. It has been shown that migration of hydrogen is much more difficult in the presence of C or O impurities than without.

The fact that hydrogen significantly reduces the vacancy formation energy that leads to a significant increase in vacancies concentration when hydrogen is absorbed in the lattice [17] and the occurrence of hydrogen-induced vacancies surrounded by hydrogen atoms (so-called superabundant vacancies) [18,19,20] also remains relevant for the efficiency of the low-energy nuclear reactions taking place in metallic environments. 

Another DFT study [21] indicates that the diffusion direction of the interstitial C and O impurities in the α-Zr is not the same—oxygen interstitials prefer diffusion along c-axis, while carbon diffuses along both a and c axes. 

Taking all of the above into account, it seems that the changing lattice structure of the target material would be a very powerful tool allowing to control nuclear reaction rates at very low energies, increasing or decreasing their yields according to the needs. 

Within this paper, we would like to explore experimentally the formation and distribution of radiation-induced defects created by D, C, and O implantation using the same Zr targets as in the previously reported UHV accelerator experiments [5]. The depth distribution of radiation-induced defects can be well observed using positron annihilation spectroscopy (PAS), which is exceptionally sensitive to open-volume defects such as vacancies, vacancy clusters, dislocations, etc., from 0.1 to 1 µm in the depth and the concentration as low as 10^−7^ [22]. In order to investigate the depth distributions of defects caused by deuterons, as well as carbon and oxygen, the variable energy positron beam (VEP) was applied, while changes in the phase of the crystal due to deuterons bombardment were examined using X-ray diffraction (XRD). 

## 2. Materials and Methods

### 2.1. Sample Preparation

Cold-rolled zirconium foils (1 mm thick; 99.2% purity) purchased from Goodfellow GmbH, Hamburg, Germany were cut into 1.5 cm × 2 cm targets. There were two unirradiated targets—one was the reference that did not undergo annealing process before the exposure in order not to destroy surface anisotropy of the crystalline structure (i.e., edge dislocations) and the defects in the target bulk. The other one was annealed at 800 °C for 3 h in vacuum conditions of 10^−5^ Torr and slowly cooled down to the room temperature. Both the reference and the annealed underwent PAS lifetime measurements (PALS) in order to compare the initial material state of the reference with the defect-free material. Samples underwent irradiation and analysis as described below.

### 2.2. Irradiation

#### 2.2.1. For PAS Measurements

**Figure 1:**
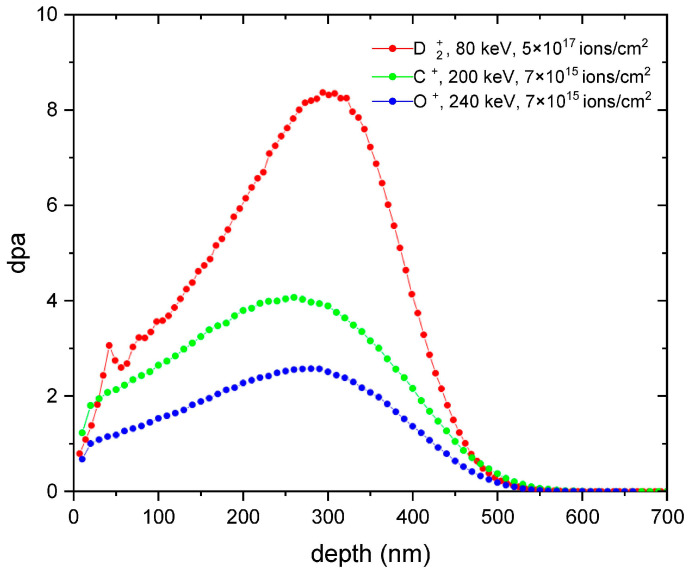
Dpa distributions obtained from SRIM/TRIM simulations of Zr target exposed to D_2_^+^, C^+^ and O^+^ ions.

Five zirconium targets (1 mm thick) of dimensions 1.5 cm × 2 cm were exposed to 80 keV D_2_^+^ beam at the ion UNIMAS 79 implantator at the Maria Curie-Skłodowska University in Lublin, Poland. Implantation dose was $5\times 10^{17}$ ions/cm^2^. Two out of the five deuterated samples were then additionally exposed to 240 keV O^+^ ions at two different doses, namely $7\times 10^{15}$ ions/cm^2^ and $5\times 10^{16}$ ions/cm^2^. Two other deuterated targets were also additionally exposed to 200 keV C^+^ ions at the same doses: $7\times 10^{15}$ ions/cm^2^ and $5\times 10^{16}$ ions/cm^2^, respectively. Beam energies of D_2_^+^, O^+^, and C^+^ were chosen in such a way that the ion ranges were roughly the same (~300–400 Å, see Figure 1). The average ion flux was $2\times 10^{14}$ cm^−2^ s^−1^ in the case of deuterons and $9.4\times 10^{12}$ cm^−2^ s^−1^ in the case of carbon and oxygen ions.

#### 2.2.2. For XRD Analysis

The same set of Zr targets was exposed at the electrostatic accelerator with ultra-high vacuum at the ELBRUS laboratory in Szczecin to the beam of 10 keV and 20 keV deuterons, which corresponds to the usually studied DD nuclear reactions. Irradiation doses were $1.25\times 10^{19}$ ions/cm^2^ and $3\times 10^{18}$ ions/cm^2^. These samples undergone XRD analysis.

### 2.3. TRIM/SRIM Simulations and Dpa Calculations

Depth distributions of the considered ions in Zr were simulated using SRIM/TRIM code in which a model with detailed calculation with full cascades was chosen [23]. Simulation’s outcome enabled to determine a widespread material radiation damage exposure unit, namely atomic displacement per atom (dpa). Dpa is the quantity that characterizes structural damages induced by a given dose of ions of given energies impinged on a particular target material.

In Figure 1, the ion depth distributions and dpa of implanted ions in Zr target are presented. The thickness of the implanted region amounts to 600 nm, while the highest concentration of defects, according to *TRIM* simulations, corresponds to the range depth of ~300 nm.

The dpa was evaluated using NRT formula [24]:

(2) $$dpa=\frac{0.8}{2E_{d}}T_{dam}\frac{ɸ}{\rho_{at}}$$

where *E_d_* corresponds to the Zr displacement energy amounting to 25 eV [25]. ɸ and *ρ_at_* are the implantation dose and the atomic density of the target (4.28 × 10^22^ atoms/cm^3^ for Zr), respectively. *T_dam_* is the damage energy which corresponds to the initial ion energy reduced by the energy dissipated in ionization, based on the e2recoil.txt, output of the SRIM/TRIM code [26]. 

### 2.4. Positron Annihilation Spectroscopy and XRD

#### 2.4.1. Positron Annihilation Lifetime Spectroscopy (PALS) 

An investigation of the initial state of the reference and annealed sample was performed using conventional positron lifetime (LT) measurements. Within this experiment, a LT spectrometer based on photomultipliers Hamamatsu H3378-50 with BaF_2_ scintillators, digital spectrometer APU8702 (TechnoAP, Ibaraki, Japan) with 180 ps time resolution was used. The positron source was the ^22^Na isotope enveloped between two 7 µm thick Kapton foils. The activity of the source was 27 µCi. The collected LT spectrum was analyzed using LT computer program [27].

#### 2.4.2. Variable Energy Positron Beam (VEP)

In order to determine the depth-distribution of defects caused by D_2_^+^, D_2_^+^ plus C^+^, and D_2_^+^ plus O^+^ ions implantation in Zr target, the variable energy positron beam (VEP) was used for PAS measurements [28,29]. In the VEP method, a flux of monoenergetic positrons is implanted with a given energy into a sample. The way to obtain a homogenous beam of slow positrons was as follows: firstly, positrons having continuous energy spectrum ranging from 0 up to 0.55 MeV was emitted by ^22^Na source. Positrons were thermalized on the frozen neon layer and then ejected with energy of several eV (due to the negative work function in solid Ne). Then, by applying voltage of +50 eV, positrons are initially accelerated. A system of magnetic fields and diaphragms is used to clear the beam with unmodulated positrons. The beam is the guided under vacuum and focused up to a diameter ~5 mm. Samples in the measuring chamber are at high potential compared to the source. Regulation of this potential establishes the energy with which positrons are implanted on the sample. Their energy can be regulated in the range from 0.05 to 36 keV. 

**Figure 2:**
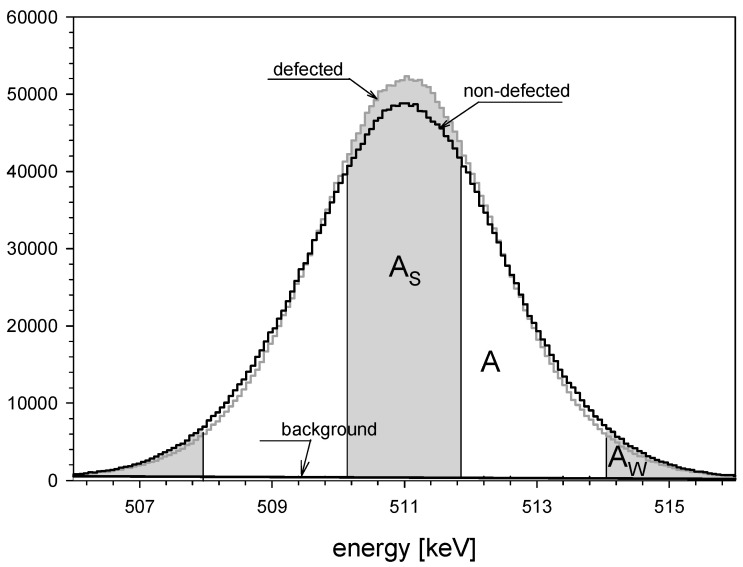
Positron annihilation lines for defected and non-defected samples with the marked areas used for the S and W parameters evaluation.

The annihilation γ-lines were recorded by a high-purity Germanium (HPGe) detector with a resolution of 1.20 keV determined for the 511 keV γ energy. The Doppler broadening of the peak reflects the longitudinal momenta of electrons moving in the direction of the detector. From the annihilation line, two shape parameters were extracted—parameter *S*, which stands for the central part of the annihilation line and *W* parameter standing for the wing part of the annihilation line (see Figure 2). The *S* parameter was calculated as a ratio of the area under central part of the peak (denoted as A_S_ in Figure 2) to the total area below the annihilation line. It defines the participation of low momenta positron–electron pair. As the momentum of the thermalized positron is negligible, momenta of positron-electron pair can be identified only as the momentum of the electron. The bigger the value of this parameter, the larger the concentration of defects in the sample. Oppositely, the *W* parameter defines the participation of high electron momenta. It was assessed as a ratio of the area below the wing part of the annihilation line (denoted as A_W_ in Figure 2) to the total area below this line. Although the Doppler-broadening spectroscopy does not provide the quantitative information about the defect sizes, the qualitative information about the change in the defect type can be obtained from the plot showing the *W* parameter as a function of *S* for values obtained for surface and saturation.

#### 2.4.3. XRD

In order to investigate the phase composition as well as the hydride formation of the zirconium targets exposed to 10 keV deuterons, X-ray diffraction—XRD (X’PERT PANalytical) with CuK_α_ radiation was used. Bragg–Brentano geometry was applied within a 2Θ angle range of 30–120°. Additionally, the surface analysis of reference and deuterated samples was carried out using the grazing incidence X-ray diffraction method. The incident angles in both cases were 3°, 5°, and 10°. X-ray beam in Zr weakened e-fold after passing 11 µm, which corresponded to the 580 nm, 960 nm, 1900 nm depth in Zr targets for incident angles 3°, 5°, and 10°, respectively. The thickness of the implanted region for 20 keV deuteron beam amounted to ~400 nm, while the highest number of particles (according to SRIM/TRIM) stopped before reaching 200 nm. 

## 3. Results

### 3.1. PALS Results

The *PALS* measurements revealed two lifetime components in the spectrum measured for the reference target. The corresponding values were as follows: τ_1_ = 127 ± 7 ps and τ_2_ = 260 ± 18 ps, which indicates the occurrence of monovacancies in the reference target bulk. Their contribution to the spectrum was 84.0 ± 1.3% and 16.0 ± 1.3%, respectively. τ_1_ was lower than the value of positron lifetime for the defect-free bulk structure of Zr and represented so-called the reduced positron lifetime component. According to a simple trapping model, it informed about partial bulk annihilation (in interstitial position) [30]. In other words, non-defected areas existed in the sample. For comparison, there was only one positron lifetime found in the annealed target that amounted to 162 ± 1 ps. This value and the τ_2_ of 260 ps for monovacancies perfectly correlated with the positron lifetime of non-defected hexagonal zirconium and zirconium with monovacancies calculated using the density function theory (DFT) [31].

The lower value of τ_1_ for the reference target bulk in respect to the positron lifetime measured in the annealed sample can be explained by the two-state trapping model that took into account that number of free positrons annihilated was reduced by the number of the positrons trapped in the defects [32]. This result indicates that the unirradiated reference sample possessed a relatively large number of vacancies, which arose from the cold-rolling method of the sample preparation.

### 3.2. VEP Results

**Figure 3:**
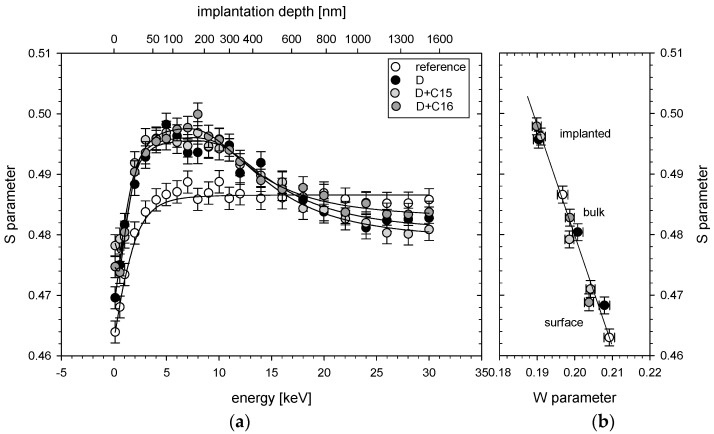
(**a**) Experimentally determined S parameters as a function of the positron incident energy in Zr targets: reference—unirradiated Zr target, D—Zr target exposed to 80 keV D_2_^+^ beam (5 × 10^17^ ions/cm^2^), D + C15—Zr target exposed to 80 keV D_2_^+^ beam (5 × 10^17^ ions/cm^2^) and 200 keV C^+^ beam (7 × 10^15^ ions/cm^2^), D + C16—Zr target exposed to 80 keV D_2_^+^ beam (5 × 10^17^ ions/cm^2^) and 200 keV C^+^ beam (5 × 10^16^ ions/cm^2^). Solid lines represent the best fit to the experimental points. (**b**) The S(W) plot based on the parameters determined using VEPFIT.

**Figure 4:**
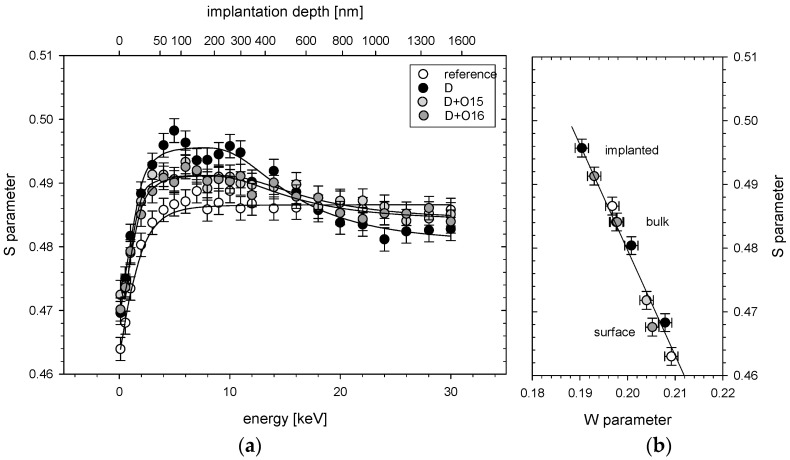
(**a**) Experimentally determined S parameters as a function of the positron incident energy in Zr targets: reference—unirradiated Zr target, D—Zr target exposed to 80 keV D_2_^+^ beam (5 × 10^17^ ions/cm^2^), D + O15—Zr target exposed to 80 keV D_2_^+^ beam (5 × 10^17^ ions/cm^2^) and 240 keV O^+^ beam (7 × 10^15^ ions/cm^2^), D + O16—Zr target exposed to 80 keV D_2_^+^ beam (5 × 10^17^ ions/cm^2^) and 240 keV O^+^ beam (5 × 10^16^ ions/cm^2^). Solid lines represent the best fit to the experimental points. (**b**) The S(W) plot based on the parameters determined using VEPFIT.

Defect profiles from VEP are depicted in Figure 3a and Figure 4a. They are displayed as the dependence of parameter S on the positron energy. On the top axis, the mean positron implantation depth in Zr, determined according to [33], is presented. In both figures, the open (white) circles represent experimental points for the reference (unirradiated) samples. Black circles correspond to the samples exposed to 80 keV D_2_^+^ beam ($5\times 10^{17}$ ions/cm^2^). Light grey circles represent deuterated Zr targets that were additionally exposed to a lower dose ($7\times 10^{15}$ ions/cm^2^) of carbon (Figure 3a) or oxygen (Figure 4a) ions and dark grey circles stay for deuterated Zr targets that were additionally exposed to higher dose ($5\times 10^{16}$ ions/cm^2^) of carbon (Figure 3a) or oxygen (Figure 4a) ions. In Figure 3b and Figure 4b, the S parameter is shown depending on the W parameter. Here, the points represent the surface of the target, the irradiated area and the bulk, corresponding to the depth which was not reached by the implanted ions. In both cases, the slope of the S(W) function was similar and did not depend on the depth, which suggests roughly the same size of all vacancies. According to the *PALS* measurements, the reference sample contained monovacancies. The same type of defects remained after the irradiation.

According to Figure 3a and Figure 4a, a large increase in the vacancy density down to the deuteron range of about 600 nm was visible for all implanted targets. For the primary used deuteron beam, the S parameter already reached its maximum value, pointing to a large number of vacancies produced. For depths above 600 nm, the S parameter was significantly lower than that obtained for the reference sample. The latter might be explained by a diffusion of the vacancies towards the sample surface as discussed in [34]. The additional carbon implantation did not increase the vacancy density either for lower or for higher carbon fluencies although the calculated dpa was much larger than for the deuteron irradiation (see Figure 1). Similar vacancy density obtained for both carbon doses may suggest a saturation of damage density resulting in a balance between creation and repair mechanisms of crystal defects that may accompany additional ion exposure, as shown in the DFT simulations [35], according to which the recombination rate of vacancies increased with the increasing vacancy concentration. In contrast, vacancy depth distribution profiles obtained for the deuterated Zr targets exposed additionally to oxygen ions showed a decrease in the S parameter (Figure 4a) compared to the deuteron irradiation for depths below 600 nm and an increase in this parameter for larger depths. Once again, we observed no dependence of the vacancy densities on the dose of implanted ions. It seems that the total number of the vacancies remained unchanged after oxygen irradiation—a reduction in their number at the target surface compared to the deuteron and carbon irradiations was aligned with the increase for the deeper layers. This behavior might be explained with a saturation of the vacancy densities, as already observed for carbon ions and diffusion of the defects towards deeper sample layers induced by oxygen irradiation. According to [16], the oxygen impurity in α-Zr has three times higher binding energy than carbon. 

By fitting the positron diffusion equation to the S(E) profiles (using the VEPFIT code [36]), the thicknesses of the defected layers at beneath the surface of the samples and the corresponding positron diffusion lengths (L_+_) have been determined. Values obtained for the reference and implanted samples are given in Table 1. The defected thicknesses presented in the table correspond well with the dpa depth profile presented in Figure 1. The diffusion lengths, however, were in all the cases much smaller than ~100 nm, which is the lower limit of the values reported for defect-free materials [37]. The L_+_ for the reference sample equal to 19 ± 4 nm indicates that the defects have already existed prior to implantation. This finding was confirmed in PALS measurements, by the LT component corresponding to monovacancies in the target bulk in the reference sample. Implantation decreased the L_+_ by a factor two in the case of all the modified targets. 

### 3.3. XRD Results

**Figure 5:**
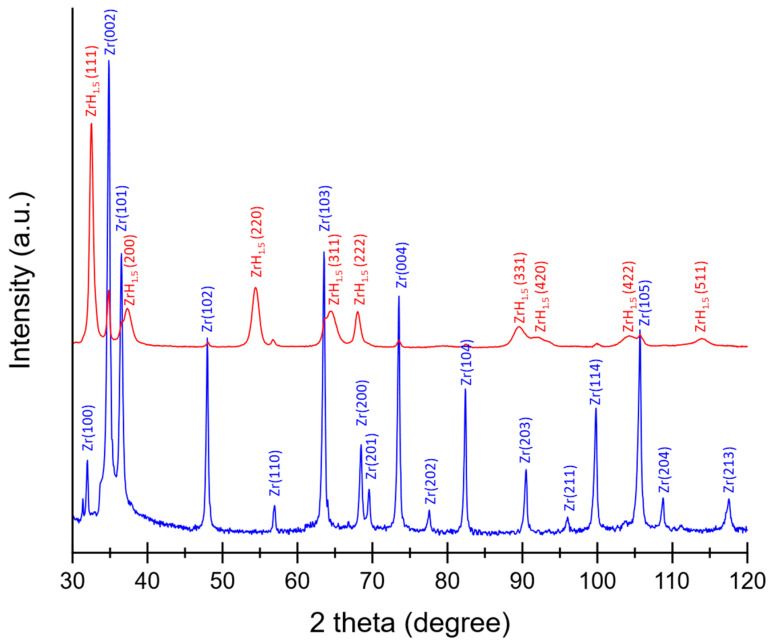
Superimposed XRD patterns of non-irradiated reference (blue line) and deuterated (red line) zirconium target.

The positron spectroscopy provides information about the defects produced within the target bulk. It is, however, not a method which can monitor the changes in phases, especially the formation of hydrides. Due to irradiation, the crystal structure of zirconium changes, the hydrides were formed, and this process may be detected by using the X-ray diffraction method. In Figure 5, a comparison of the XRD patterns of the reference and deuterated targets are presented. The acquired data were processed using X’Pert HighScore (v. 2.2.1.) software provided by Panalytical [38].

The unirradiated (reference) Zr target (see Figure 1) had a preferred (002) orientation. Together with the (100), (101), (102), and (110) sequence of peaks, the diffraction spectrum of Zr sample confirmed the hexagonal close-packed (hcp) crystal structure. In the XRD of the deuterated sample, additional diffraction peaks, i.e., (111), (200), (220), and (311), corresponding to the face cantered cubic structure of the zirconium deuteride ZrH_1.5_ were present. At the deuterium concentration represented by the ZrH_1.5_ stoichiometry (δ-phase), the deuterium occupied tetrahedral positions.

Stoichiometry of the zirconium hydrides determined in the nuclear experiments should amounted to 2.0. Lower values (1.5–1.66) observed in the XRD indicates binding of deuterium by vacancies. It has already been shown that up to nine hydrogen atoms can be trapped by a single vacancy [16]. 

In particular, DFT simulations showed that irradiation induces anisotropic expansion of the lattice parameter *a* of the hcp crystal structure and contraction of the *c* parameter [34]. Our results for deuterated Zr targets confirmed this finding. The values of the lattice parameters of unexposed Zr targets amounted to: *a* = 5.168 Å and *c* = 3.213 Å and changed after the exposure as follows: *a* = 5.177 Å and *c* = 3.203 Å.

**Figure 6:**
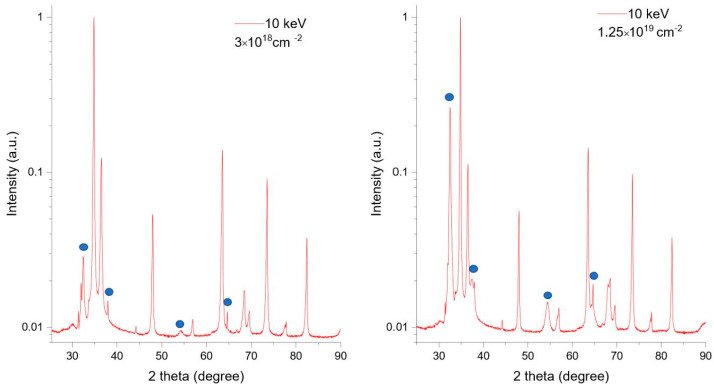
XRD patterns of Zr targets exposed 10 keV deuterons of two different doses: 3 × 10^18^ deuterons/cm^2^ and 1.25 × 10^19^ deuterons/cm^2^.

In Figure 6, two different XRD patterns obtained after exposure of the reference Zr target to two different doses ($3\times 10^{18}$ deuterons/cm^2^ and $1.25\times 10^{19}$ deuterons/cm^2^) of 10 keV deuteron beam are presented. Peaks corresponding to hydrides are marked by circles. They corresponded to lattice planes (111), (200), (220), and (311), respectively. According to Figure 6, it is visible that the intensities of the peaks corresponding to zirconium hydrides increased with the increasing fluence. 

**Figure 7:**
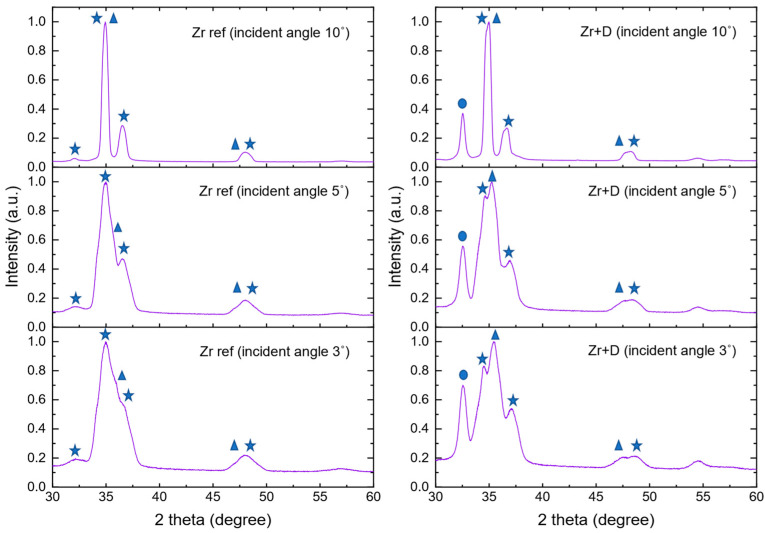
XRD patterns obtained using grazing angle incidence diffraction method at three different incident angles: 3°, 5°, and 10°. On the left side, the diffraction patterns obtained for the reference target are presented. On the right side—for the deuterated target. The marked peaks correspond to: ZrH_1.6_ (circles), Zr_3_O (triangles), Zr (stars).

A more detailed XRD analysis of the surface of the reference and deuterated sample was performed by applying the grazing incidence X-ray diffraction method. The experimental patterns were deconvoluted by means of Gaussian function using Origin2021b software. In Figure 7, the positions of deconvoluted peaks, corresponding to zirconium (100), (002), (101), and (102) planes, zirconium deuteride ZrD_1.66_ (111) plane, and zirconium oxide Zr_3_O (101) and (102) planes are marked. The analysis focused on the ratio of the area under the most intensive peaks corresponding zirconium oxide and zirconium (Zr_3_O/Zr) as well as zirconium deuteride and zirconium (ZrD_1.66_/Zr). In the case of the deuterated sample, the ratio of peak areas under the most intensive oxide peak to the most intensive zirconium peak, measured for incident angle ω = 3°, 5°, and 10°, amounted to 0.88 ± 0.03, 0.52 ± 0.02 and 0.77 ± 0.02, respectively. The ratio of deuterides to zirconium was 0.49 ± 0.01, 0.31 ± 0.01 and 0.45 ± 0.01. The deconvolution of the reference non-deuterated sample revealed that the intensities of the oxide peaks were much lower-almost negligible. This finding agrees to a usual very thin oxidation layer of Zr of ~2–3 nm [39,40]. which is much less than the range of X-rays in Zr. In the case of the deuterated sample, the oxide peaks were much more intensive regardless of the incident angle, which is an indication that irradiation imposes movement of the oxygen into the deeper layers of the Zr target. Similarly, the significant intensities of the hydride peak measured at all the incident angles proves that implanted deuterons diffuse into the bulk.

**Figure 8:**
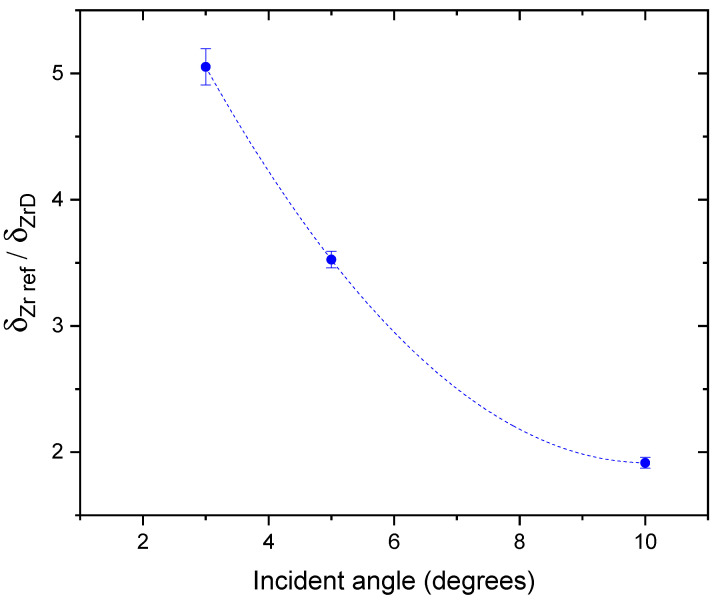
Ratio of the dislocation density of the reference and deuterated sample at three different incident angles: 3°, 5°, and 10°. The dotted line is drawn to guide the eye.

Deconvoluted peaks were additionally used to determine the density of dislocations *δ* as the reciprocal of the square of the crystallite size *D* obtained from the Scherrer’s equation for the line broadening of the peak: $D=\left(k\;\lambda \right)/\left(\beta \;cos\theta \right)$. Here, *λ* is the wavelength of the X-ray, *β* corresponds to the FWHM width of the diffraction peak, *θ* is the diffraction angle, and *k* is the shape factor constant. Consequently, the dislocation density can be obtained from the equation: $\delta =1/D^{2}.$ The method used here was very simple and can be applied only for general characterization of observed structural changes. Therefore, to compare reference and deuterated samples, we calculated only the ratio δ_Zr ref_/δ_ZrD_, which reduces systematic uncertainties of the results, such as the instrumental line width. In Figure 8, the ratios obtained for three different incident angles are presented. Regardless of the target, the closer to the surface, the higher the dislocation density (greater line widths) was observed, which is not unusual due to the mechanical stress caused by the cold rolling of the samples. The density of dislocations, however, was much larger in the reference target than in the deuterated one, which corresponds to the smaller crystallite size. According to Figure 8, the δ_Zr ref_/δ_ZrD_ ratio decreased with the increasing incident angle (3°, 5° and 10°) and amounted to 5.05 ± 0.14, 3.52 ± 0.07 and 1.92 ± 0.04, respectively. This means that the observed changes were limited to the surface region of the sample.

## 4. Discussion and Conclusions

Nuclear reaction cross sections at very low projectile energies taking place in metallic media strongly depend on the effective electron mass due to the electron screening of the involved ion charges. Thus, the crystal lattice defects induced by ion irradiation of the target samples are probably responsible for the observed enhancement of the screening energies in the deuteron–deuteron fusion reactions in accelerator experiments on Zr targets [5]. In the present paper, a role of dislocations and creation of vacancies in the deuteron irradiated targets under influence of carbon and oxygen contaminations has been studied in detail. Two different spectroscopic methods, positron annihilation spectroscopy (PAS) and X-ray diffraction (XRD), which are sensitive to the crystal vacancies and dislocations, respectively, have been applied to distinguish their contribution in nuclear reactions. The first method shows that a deuteron beam can produce stable vacancies in the Zr target samples distributed in agreement with the expected dpa depth profile. A signature for a diffusion of vacancies towards surface has been found when comparing with non-irradiated samples. The role of carbon and oxide contaminations has been tested by means of implantation of corresponding ions of adjusted energies into Zr samples at two different doses. In the case of carbon implantation, no change of the vacancy depth distribution produced by deuteron irradiation has been observed. This suggests that the carbon ions which are expected to produce much more dpa’s that deuterons do not change the total number of vacancies as well. Consequently, we can speculate that the number of vacancies produced by deuterons is so high that creation of new vacancies by carbon ions is balanced by their repair mechanisms, and the implanted carbon ions itself are bound to the existing vacancies together with deuteron atoms. The situation is different in the case of oxygen ion implantation. Although the depth distribution of vacancies is similar for two different implantation doses, it is strongly changed when compared to the reference sample implanted only with deuterons. A reduction in the *S* parameter was observed in the region below 600 nm, corresponding to the range of implanted ions, and increased in the deeper region. Since the total number of vacancies seemed not to be changed, we can conclude that original vacancies produced by deuterons were diffused to deeper sample regions. Furthermore, similar to the carbon case, there was a balance between production of new vacancies and their annihilation. Thus, the oxygen atoms probably coupled to the earlier existing vacancies. The different mobility of carbon and oxygen vacancies can be then explained by their different binding energies in the Zr crystal lattice. While the carbon atoms should have a large binding energy to the deuteron-produced vacancies (D-C-Vacancy structure) and even increase their binding energy in Zr reducing vacancy diffusion, the oxygen atoms have also a large vacancy binding energy (D-O-V structure) but reduce their binding energy in lattice allowing for easier diffusion, which is in agreement with DFT calculations [16]. However, in both cases, the vacancy conglomerates should have a similar size, as presented in the *S* vs. *W* diagrams in Figure 3 and Figure 4.

The diffusion of oxygen atoms to deeper layers could also be confirmed in the XRD analysis performed at different grazing angles. The determined deuteron-metal stoichiometry ZrD_1.66_ of studied samples agrees with the observed increase in the lattice constant, but on the other hand, it is much lower than that determined in the DD fusion reaction of D/Zr = 2/1 [1,2]. The latter probably reflects binding of some deuteron atoms by vacancies, which XRD is not sensitive to. The density of crystal dislocations observed in the samples is related to broadening of the XRD lines, and, therefore, to the crystallite size. The analysis showed that the dislocation density (crystallite size) was the highest (lowest) for the reference sample and decreased (increased) with the depth (see Figure 8), which is due to cold-rolling procedure of virgin samples. The dislocations density at the surface of the deuterated samples determined from crystallite sizes was much lower than for the reference target. Such correlation between the increase in the grain size due to irradiation and reduction in the dislocation density was reported in [41,42]. On the other hand, we observed a decrease in the diffusion lengths for deuterated samples determined in the VEP analysis (see Table 1), which should correspond to an increase in the defect density at the surface. The latter can probably be explained by much higher sensitivity of PAS for vacancies than dislocations. The cold-rolling procedure was also responsible for the relatively high density of vacancies in deeper layers of the reference sample up to 1600 nm (Figure 3 and Figure 4). 

In conclusion, the experimental results obtained in this work show that deuteron irradiation of Zr targets leads to high vacancy densities, which is responsible for the increase in the effective electron mass and the corresponding enhancement of the electron-screening effect observed in the DD reactions at very low deuteron energies. This is why we observed an increase of the reaction yield under ultra-high vacuum conditions in the first hours after the cleaning the Zr target by means of argon sputtering [43]. Possible carbon and oxygen impurities occurring on the Zr target surface during the deuteron irradiation can influence nuclear reactions additionally. Carbon ions do not change the absolute number and the depth distribution of the vacancy density in the Zr target. On the other hand, oxygen ions reduce the number of vacancies at the Zr target surface and induce their diffusion to deeper target layers. In this way, the observed DD reaction rate might be reduced during the deuteron irradiation time, leading to a maximum of the reaction yield after two–three hours. The role of individual impurities is planned to be investigated in next experiments under a controlled implantation process.

## Figures and Tables

**Table 1:** Thicknesses of defected layers and L_+_ after implantation to: D_2_^+^ beam (5 × 10^17^ ions/cm^2^) and additionally to C^+^ or O^+^ beam of two different fluencies: 7 × 10^15^ ions/cm^2^ (described as O15 or C15) and 5 × 10^16^ ions/cm^2^ (described as O16 or C16).

Target	Reference	D_2_^+^	D_2_^+^ + C15	D_2_^+^ + C16	D_2_^+^ + O15	D_2_^+^ + O16
Thickness of defected the layer [nm]	-	504 ± 42	572 ± 50	530 ± 48	576 ± 60	541 ± 57
L_+_ [nm]	19 ± 4	9 ± 3	10 ± 3	10 ± 3	10 ± 3	11 ± 3

## Data Availability

Research data is available upon request.
